# Reducing the risk of suicidal behaviors in medical graduate students: exploration of student-supervisor relationship and subjective family socioeconomic status

**DOI:** 10.3389/fpsyt.2024.1381291

**Published:** 2024-05-07

**Authors:** Yan Wu, Zheng Qu, Wanjie Tang, Yunhao Zheng, Xin Xiong, Zheng Ye, Zhenlin Li

**Affiliations:** ^1^ College of Marxism, Sichuan University, Chengdu, China; ^2^ Department of Postgraduate Students, West China School of Medicine/West China Hospital, Sichuan University, Chengdu, China; ^3^ School of Allied Health Sciences, West China School of Medicine/West China Hospital, Sichuan University, Chengdu, China; ^4^ Center for Health and Psychology, Sichuan University, Chengdu, China; ^5^ West China Hospital of Stomatology, Sichuan University, Chengdu, China; ^6^ Department of Radiology, West China Hospital, Sichuan University, Chengdu, China

**Keywords:** suicidal behaviors, medical graduate student, student-supervisor relationship, subjective family socioeconomic status, medical education, preventive strategy

## Abstract

**Objective:**

This study aimed to investigate the association between the risk of suicidal behaviors and student-supervisor relationships and subjective family socioeconomic status (SFSS) in medical graduate students, and to propose preventive strategies to reduce the suicidal risk among medical graduate students.

**Materials and methods:**

A total of 1,310 validated questionnaires were collected from medical graduate students, which included demographic information, study programs, the Suicidal Behaviors Questionnaire-Revised (SBQ-R) questionnaire, the Leader-Member Exchange 7 (LMX-7) questionnaire, and SFSS by MacArthur Scale. Multiple regression analysis was employed to examine the associations between variables and adjust for confounders. A moderation analysis, containing simple slope analysis and Johnson-Neyman interval plots were used to analyze the moderating effect of the SFSS in the association of SBQ-R and LMX-7 scores.

**Results:**

A total of 88 participants (6.7%) were at risk of suicidal behaviors. In the high-quality student-supervisor relationship group (LMX-7 score ≥ 25), SFSS was significantly higher than in the low- and moderate-quality relationship group (p=0.002). The median SBQ-R score and proportion of suicide risk was significantly lower (p<0.001) in the high-quality student-supervisor relationship group. Multiple regression analysis indicated LMX-7 scores (β=-0.098, 95% CI [-0.118, -0.077], p<0.001) and SFSS (β=-0.073, 95% CI [-0.127, -0.019], p=0.008) were significantly negatively associated with SBQ-R, whereas the interaction term of SFSS with LMX-7 (β=0.018, 95% CI [0.007, 0.029], p=0.001) showed a significant positive association with SBQ-R. The Johnson-Neyman interval showed a significant association between LMX-7 and SBQ-R scores only when SFSS was less than 7.82 (p<0.05).

**Conclusion:**

The risk of suicidal behaviors was associated with student-supervisor relationships and SFSS among medical graduate students. Poor relationships with supervisor were associated with an elevated risk of suicidality, and SFSS moderated this association. Educators should pay increased attention to the suicidal risk of medical graduate students with poor supervisor relationships, especially those from families with low SFSS, and provide timely preventive strategies.

## 1 Introduction

Suicide is a serious public health issue and a significant concern, especially in the field of medical education (1). Due to the demanding nature of medical study, medical students often experience higher levels of stress, anxiety, depression, burnout, and mental disorders than the general population (2, 3). Recent statistics indicate that suicidal ideation is prevalent among medical students, with rates ranging from 6.0% to 43.0% (4), and is considered as a strong predictor of suicide attempts (5). In addition, medical graduate students, struggling with the pressure from a variety of academic, personal, and environmental challenges, may be at an elevated risk for suicide behaviors (6, 7). For example, unpredictable study duration or delayed graduation, financial insecurity, unstable relationships, competitive job markets, may trigger negative feelings and put them at higher risk of suicide.

Throughout the journey of pursuing master or doctoral degrees, medical graduate students greatly rely on supervisory support, both academically and emotionally. Previous studies showed that student-supervisor relationship can directly affect the creativity and decision-making skills of students (8–10), as well as their subjective well-being and psychological safety (11, 12). To build a healthy student-supervisor relationship, supervisors are supposed to offer students with academic support (e.g. instruction and mentoring) and emotional support (e.g. trust and encouragement). However, in Asian education systems based on Confucianism, such as China, the student-supervisor relationship is often a supervisor-centered, top-down hierarchical relationship (11). Under such educational environments, medical graduate students can hardly have effective and equal communications with their supervisors, which may result in supervisors being unable to provide appropriate academic guidance. Moreover, it is difficult for medical graduate students to have emotional interactions in this student-supervisor relationship, which may further cause mental disorders of students, and may even lead to suicidal ideation and suicidal behaviors.

As suicide is a complex multicausal phenomenon, it requires investigations from different perspectives, including health, educational, psychological, and socioeconomic factors (13). Several studies have explored the association between suicidality and socioeconomic factors, and found that socioeconomic factors are one of the determinants of suicidal behaviors (14, 15). Likewise, low socioeconomic status has been found to be associated with suicidal ideation among college students (16). At the individual level, low income has been considered as a risk factor for suicidal behaviors among medical students (17). At the population level, suicide rates among medical students in low- and middle-income countries were significantly higher than those in high-income countries (18). Furthermore, recent studies have shown that the perceived family socioeconomic status, also known as subjective family socioeconomic status (SFSS), plays a moderating role in self-injury relationship among medical students (19), and may attenuate the association between suicidal ideation and suicide attempts.

Therefore, in this study we hypothesized that the risk of suicidal behaviors would be associated with student-supervisor relationships and SFSS among medical graduate students, and that SFSS would play a moderating role in it.

## 2 Materials and methods

### 2.1 Study participants and data collection

The current study was reviewed and approved by the local ethics committee (registration number: 2023-2186), and all participants provided informed consent. The self-report questionnaire was distributed to three medical colleges from the largest comprehensive medical school in western China, including medical graduate students majoring in clinical medicine, medical technology, and nursing. The questionnaire link was sent to individuals via email and mobile phone text messages, and a scientific research coordinator followed the completion of the questionnaires. A total of 1,310 valid questionnaires were returned from 1,316 distributed questionnaires, with an effective rate of 99.5%. Demographic information, including age, gender, marital status, study program, and history of mental illness, as well as specialized scales information were collected for all participants.

### 2.2 Measures

#### 2.2.1 Risk of suicidal behaviors

The Suicidal Behaviors Questionnaire-Revised (SBQ-R) questionnaire was utilized to evaluate the risk of suicidal behaviors in medical graduate students. The SBQ-R questionnaire comprises of four items assessing previous suicidal ideation/attempts, the frequency of suicidal ideation, the threat of suicidal attempts, and the future likelihood of suicidal behaviors (**Supplementary Material**). The SBQ-R is scored on a scale of 3 to 18, with higher scores indicating a higher risk of suicidal behaviors. According to previous studies, the cut-off value for suicide risk was 7 in the general adult population (20). Therefore, the participants were divided into those with suicidal risk (SBQ-R scores ≥ 7) and without significant suicidal risk (SBQ-R scores < 7) in this study.

#### 2.2.2 Student-supervisor relationship

The quality of the relationships between medical graduate students and their supervisors was assessed using the Leader-Member Exchange 7 (LMX-7) questionnaire. The leader-member exchange relationship originally refers to the exchange relationship between a leader and a subordinate, which is similar to that between a supervisor and a graduate student in China. The LMX-7 provides a quantitative measure of mutual respect, trust, and expectations of such exchange relationship, and its reliability and validity have been well-established in evaluating student and supervisor relationships (8, 9).

The LMX-7 includes seven items relating to student perceptions of the relationship (**Supplementary Material**). A sample item is “How well does your supervisor recognize your potential?”. The LMX-7 is based on a 5-point Likert scale, and these items were rated from 1 to 5 (larger number indicating higher degree or possibility). The total score ranges from 5 to 35, with higher scores indicating a better relationship quality. Typically, a score of 25 or higher is indicative of a high-quality relationship (21).

#### 2.2.3 Subjective family socioeconomic status

The SFSS of participants was measured by MacArthur Scale (22), which is a ladder-based survey tool to assess their relative status on several domains of family socioeconomic status and their subjective overall position (**Supplementary Material**). In brief, participants were asked to choose their position on a 10-step ladder representing diffident position of their family, with the bottom ladder (step 1) representing the worst living conditions, the lowest education level, the least decent jobs, and the lowest incomes, while the top ladder (step 10) representing the best living conditions, the highest education level, the most decent jobs, and the highest incomes. The scale has been widely used in studies and has shown good reliability.

### 2.3 Statistical analysis

Cronbach’s coefficient was employed to evaluate the questionnaire’s reliability, and the bootstrap method was utilized to estimate its 95% confidence interval (CI). The continuous variables were presented as means and standard deviations (SD) or median and interquartile ranges (IQR), according to their normality, which was assessed by the Shapiro-Wilk test. The Student’s t-test was used for comparing normally distributed variables, and the Wilcoxon rank sum test was used for comparing non-normal variables. The categorical variables were presented as numbers and percentages, and compared by using chi-square test. Multiple linear regression models were used to analyze the associations between variables and to adjust for confounders, and LMX-7 scores and SFSS were centered in the regression analysis. A moderation analysis was conducted to examine the moderating effect of SFSS on the association between student-supervisor relationships and suicidality. Subgroup analyses were used to analyze differences in effects between males and females. All statistical analyses were conducted in R (version 4.2.2, The R Foundation for Statistical Computing), and the significance level α was set to 0.05.

## 3 Results

### 3.1 Participants information

**Figure 1:**
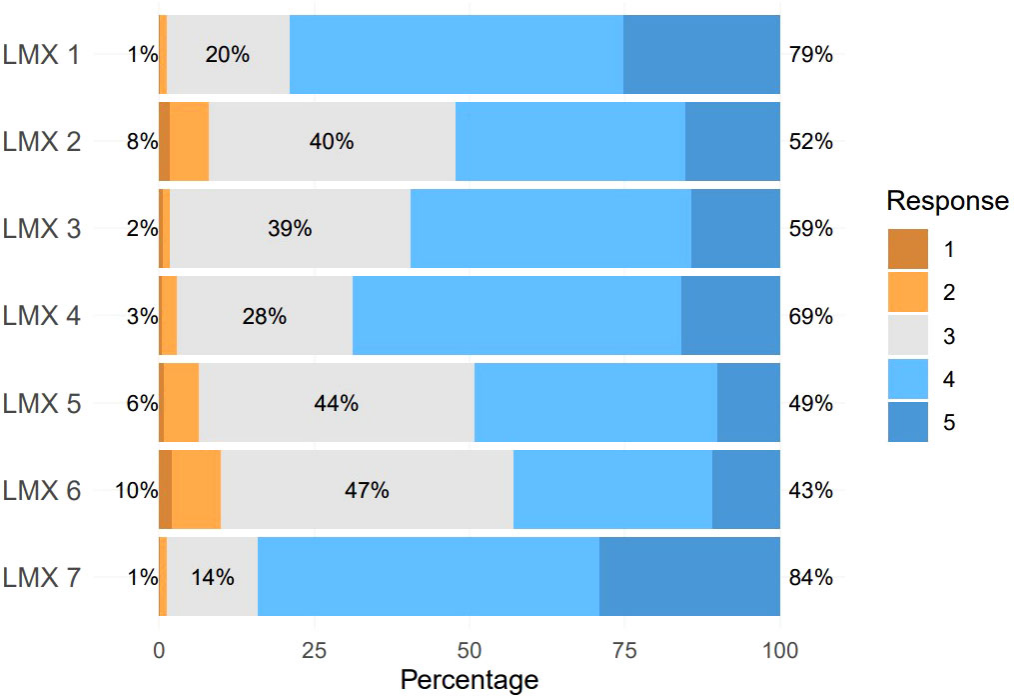
Distribution of the scores for each item of the LMX-7 questionnaire. Left percentage numbers indicate response 1 and response 2, middle percentage numbers indicate response 3, right percentage numbers indicate response 4 and response 5.

The participants included 471 males (36.0%) and 839 females (64.0%), with a median age of 25.5 years (IQR: [24.0, 28.0]). Among these participants, 231 were married (17.6%), 750 were in master’s programs (57.3%), and 560 were in doctoral programs (42.7%). The median scores for LMX-7 and SBQ-R were 26.0 (IQR: [23.0, 28.0]) and 3.0 (IQR: [3.0, 4.0]). The median SFSS level was 5.0 (IQR: [4.0, 6.0]). With a cutoff score of 7 on the SBQ-R score, a total of 88 participants (6.7%) were at risk of suicide. A total of 18 participants had a history of mental illness (1.4%). **Figure 1** illustrates the distribution of each item of the LMX-7 questionnaire. The Cronbach’s coefficient was 0.900 (95% CI: 0.889, 0.909) for the LMX-7 and 0.774 (95% CI: 0.723, 0.817) for the SBQ-R.

As demonstrated in **Table 1**, with the cut off score of 25, a total of 850 participants had a high-quality relationship with their supervisors (64.9%), while 460 participants had a low- or moderate-quality relationship with their supervisors (35.1%). In the high-quality relationship group, the median age (p=0.001), proportion of males (p<0.001), proportion of doctoral programs (p=0.003), SFSS (p=0.002), and proportional of marriage status (p=0.012) were significantly higher compared to the low- and moderate-quality relationship group. Conversely, the median SBQ-R score and the risk of suicide behaviors was significantly lower in the high-quality relationship group (p<0.001).

**Table 1:** Comparison of the low- and moderate-quality student-supervisor relationship group and the high-quality student-supervisor relationship group.

Variable	LMX-7 ≤ 24		LMX-7 ≥ 25		*p*
	Mean (SD)	Median [IQR]	Mean (SD)	Median [IQR]	
**N (%)**	460 (35.1)		850 (64.9)		
**Age**	25.9 (3.3)	25.0 [24.0, 27.2]	26.6 (3.8)	26.0 [24.0, 28.0]	0.001
Sex (%)					<0.001
Male	135 (29.3)		336 (39.5)		
Female	325 (70.7)		514 (60.5)		
Program (%)					0.003
Master	289 (62.8)		461 (54.2)		
Doctor	171 (37.2)		389 (45.8)		
**SFSS**	4.6 (1.6)	5.0 [3.0, 5.0]	4.8 (1.6)	5.0 [4.0, 6.0]	0.002
Marriage (%)					0.012
Yes	64 (13.9)		167 (19.6)		
No	396 (86.1)		683 (80.4)		
**LMX-7 Score**	21.8 (2.2)	22.0 [21.0, 23.0]	28.6 (3.0)	28.0 [26.0, 30.0]	<0.001
LMX 1	3.4 (0.6)	3.0 [3.0, 4.0]	4.4 (0.5)	4.0 [4.0, 5.0]	<0.001
LMX 2	2.9 (0.7)	3.0 [3.0, 3.0]	3.9 (0.8)	4.0 [3.0, 4.0]	<0.001
LMX 3	3.0 (0.4)	3.0 [3.0, 3.0]	4.1 (0.6)	4.0 [4.0, 4.0]	<0.001
LMX 4	3.1 (0.6)	3.0 [3.0, 3.0]	4.2 (0.5)	4.0 [4.0, 4.0]	<0.001
LMX 5	2.9 (0.5)	3.0 [3.0, 3.0]	3.9 (0.7)	4.0 [3.0, 4.0]	<0.001
LMX 6	2.8 (0.6)	3.0 [3.0, 3.0]	3.8 (0.8)	4.0 [3.0, 4.0]	<0.001
LMX 7	3.6 (0.7)	4.0 [3.0, 4.0]	4.4 (0.5)	4.0 [4.0, 5.0]	<0.001
**SBQ-R Score**	4.3 (2.2)	3.0 [3.0, 5.0]	3.5 (1.2)	3.0 [3.0, 3.0]	<0.001
SBQ-R 1	1.2 (0.5)	1.0 [1.0, 1.0]	1.1 (0.3)	1.0 [1.0, 1.0]	<0.001
SBQ-R 2	1.2 (0.6)	1.0 [1.0, 1.0]	1.0 (0.3)	1.0 [1.0, 1.0]	<0.001
SBQ-R 3	1.1 (0.3)	1.0 [1.0, 1.0]	1.0 (0.2)	1.0 [1.0, 1.0]	<0.001
SBQ-R 4	0.8 (1.3)	0.0 [0.0, 2.0]	0.4 (0.9)	0.0 [0.0, 0.0]	<0.001
History of Mental Illness (%)					0.929
No	453 (98.5)		839 (98.7)		
Yes	7 (1.5)		11 (1.3)		
Suicide Risk (%)					<0.001
Yes	58 (12.6)		30 (3.5)		
No	402 (87.4)		820 (96.5)		

The Wilcoxon rank sum test was used to compare non-normal continuous variables; the chi-square test was used to compare categorical variables. SD, standard deviation; IQR, interquartile range. LMX, leader-member exchange; SFSS, subjective family socioeconomic status.

### 3.2 Multiple regression analysis and moderation analysis

**Figure 2:**
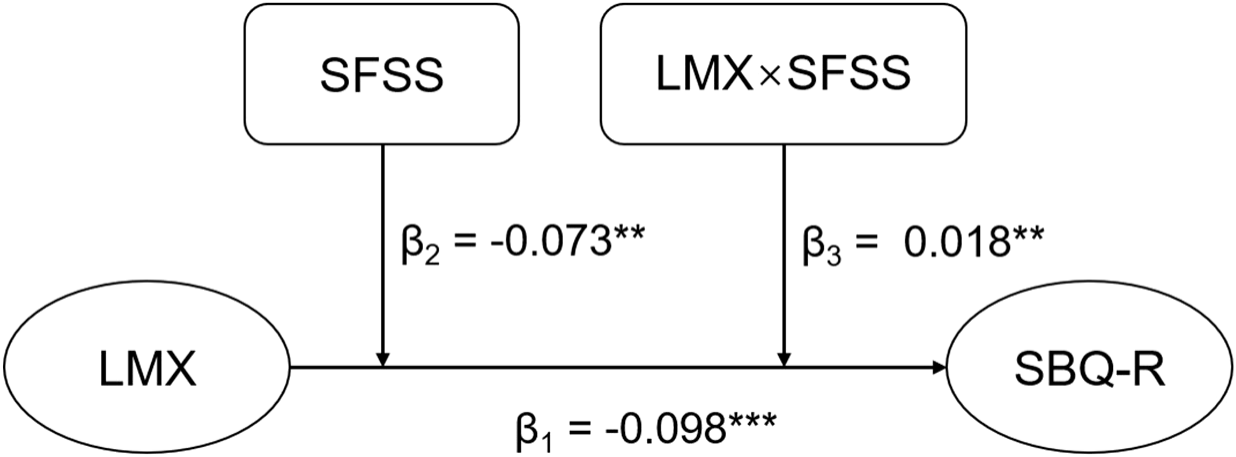
The moderating effect of SFSS. **p<0.01, ***p<0.001. LMX, leader-member exchange; SFSS, subjective family socioeconomic status.

As shown in **Table 2**; **Figure 2**, multiple regression models indicated a significant negative association between centered LMX-7 and SBQ-R scores (β=-0.103, 95% CI [-0.123, -0.082], p<0.001), controlling for age, gender, program, marital status, and history of mental illness. In the model that included SFSS and an interaction term for SFSS with LMX-7 scores additionally, LMX-7 scores (β=-0.098, 95% CI [-0.118, -0.077], p<0.001) and SFSS (β=-0.073, 95% CI [-0.127, -0.019], p=0.008) were significantly negatively associated with SBQ-R, whereas the interaction term of SFSS with LMX-7 (β=0.018, 95% CI [0.007, 0.029], p=0.001) showed a significant positive association with SBQ-R.

**Table 2:** Multiple regression analysis and moderation analysis for SBQ-R scores.

Variable	Model 1	Model 2	
Age	0.006	0.001	
	(-0.029, 0.041)	(-0.034, 0.036)	
Sex			
Male	Reference	Reference	
Female	0.04	0.058	
	(-0.143, 0.223)	(-0.125, 0.240)	
Program			
Master	Reference	Reference	
Doctor	-0.244*	-0.237*	
	**(-0.466, -0.022)**	**(-0.458, -0.016)**	
Marriage			
Yes	Reference	Reference	
No	-0.046	-0.058	
	(-0.336, 0.243)	(-0.347, 0.230)	
Marriage			
No	Reference	Reference	
Yes	2.550***	2.553***	
	**(1.807, 3.292)**	**(1.815, 3.292)**	
LMX-7 Score	-0.103***	-0.098***	
	**(-0.123, -0.082)**	**(-0.118, -0.077)**	
SFSS		-0.073**	
		**(-0.127, -0.019)**	
SFSS × LMX-7 Score		0.018**	
		**(0.007, 0.029)**	

β (95% CI). *p<0.05, **p<0.01, ***p<0.001. The bold indicates p<0.05. LMX, leader-member exchange; SFSS, subjective family socioeconomic status.

**Figure 3:**
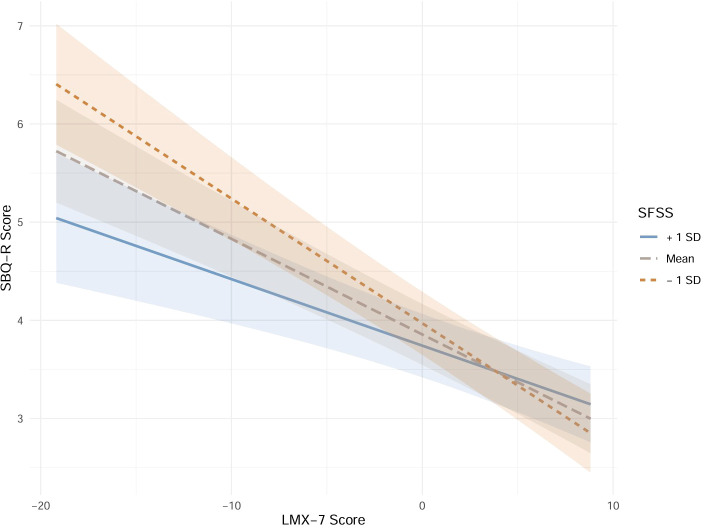
The simple slope plots. SD, standard deviation; LMX, leader-member exchange; SFSS, subjective family socioeconomic status.

As illustrated in **Figure 3**, simple slope analysis indicated that the effect of LMX-7 scores on SBQ-R scores varied across different SFSS groups. In the high SFSS group (+1 Standard deviation), the slope was -0.068, which was smaller than the slope in the mean SFSS group (Slope = -0.098). Furthermore, the effect of LMX-7 scores on SBQ-R scores in the mean SFSS group was smaller than that in the low SFSS group (-1 Standard deviation, Slope = -0.127).

**Figure 4:**
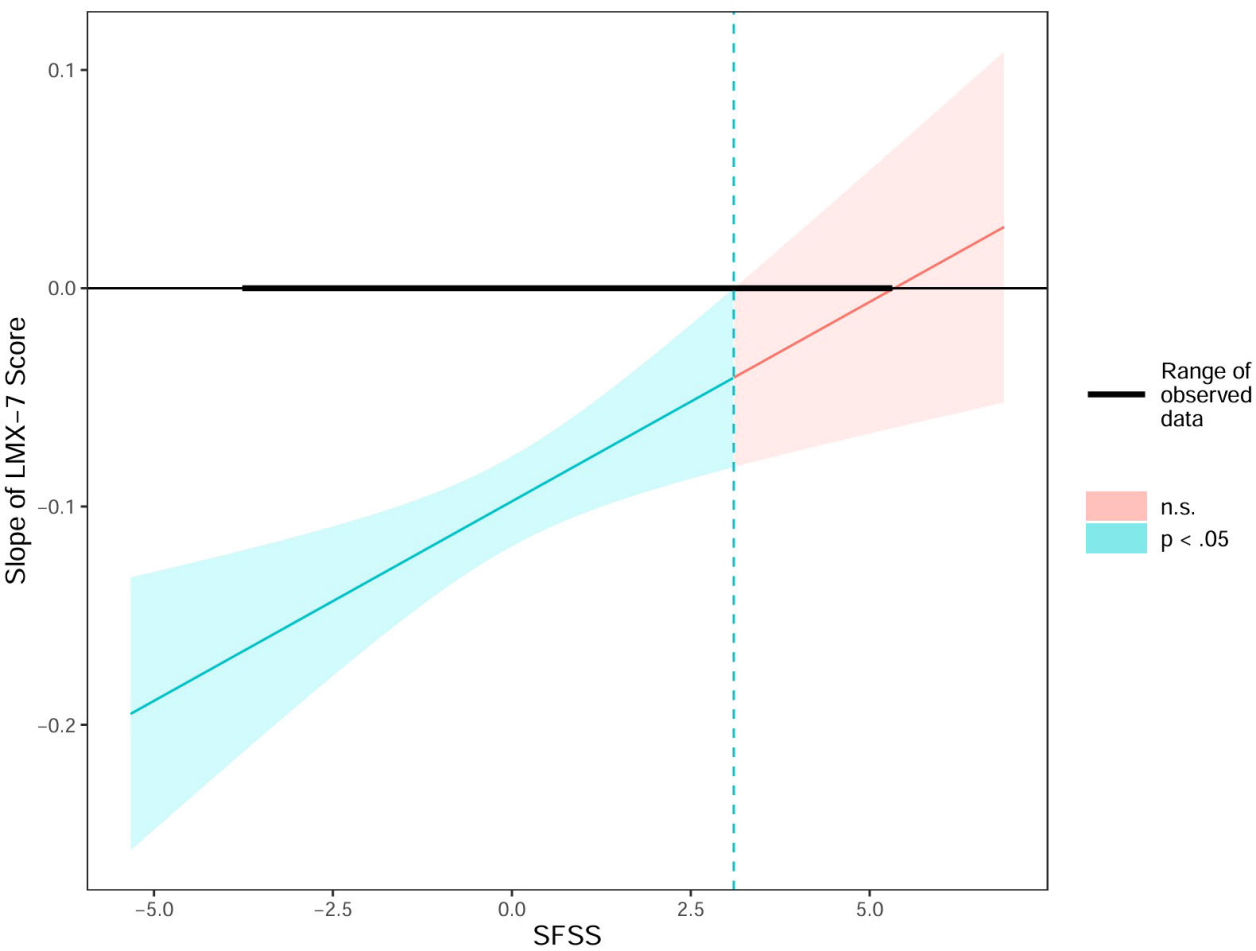
The Johnson-Neyman interval plot. LMX, leader-member exchange; SFSS, subjective family socioeconomic status; n.s, no significance.

In addition, the Johnson-Neyman interval plot (**Figure 4**) showed a significant association between LMX-7 scores and SBQ-R scores when the centered SFSS was below 3.10 (i.e., when the SFSS was less than 7.82, p<0.05). Conversely, the association was not significant (p ≥ 0.05) when the SFSS was greater than 7.82.

### 3.3 Subgroup analysis

Subgroup analyses revealed that male medical graduate students had significantly higher median ages (p<0.001), percentages of doctoral program enrollment (p<0.001), and LMX-7 scores (p<0.001) than females. Conversely, their marriage rates (p=0.042) and SBQ-R scores (p=0.045) were significantly lower than those of females (**Table 3**). Additionally, no significant differences were observed in SFSS, history of mental illness, or suicide risk between male and female students (p>0.05). Multiple linear regression showed that the effect of SFSS on SBQ-R scores and its moderating effect on the association between LMX-7 and SBQ-R scores were more pronounced in female medical graduate students than in the male medical graduate student population (**Table 4**). In contrast, among male medical graduate students, neither the estimated effects of SFSS on SBQ-R scores nor its moderating effects reached statistical significance (p>0.05).

**Table 3:** Comparison of the male and the female group.

Variable	Male		Female		p
	Mean (SD)	Median [IQR]	Mean (SD)	Median [IQR]	
**N**	471 (36.0)		839 (64.0)		
**Age**	27.0 (3.7)	26.0 [24.0, 29.0]	26.0 (3.5)	25.0 [24.0, 27.0]	<0.001
Degree (%)					<0.001
Master	235 (49.9)		515 (61.4)		
Doctor	236 (50.1)		324 (38.6)		
**SFSS**	4.6 (1.8)	5.0 [3.0, 6.0]	4.8 (1.5)	5.0 [4.0, 6.0]	0.071
Marriage (%)					0.042
No	97 (20.6)		134 (16.0)		
Yes	374 (79.4)		705 (84.0)		
**LMX-7 Score**	27.0 (4.6)	27.0 [24.0, 29.0]	25.7 (4.0)	26.0 [23.0, 28.0]	<0.001
LMX 1	4.1 (0.7)	4.0 [4.0, 5.0]	4.0 (0.7)	4.0 [4.0, 4.0]	<0.001
LMX 2	3.8 (0.9)	4.0 [3.0, 4.0]	3.5 (0.9)	3.0 [3.0, 4.0]	<0.001
LMX 3	3.8 (0.8)	4.0 [3.0, 4.0]	3.6 (0.7)	4.0 [3.0, 4.0]	<0.001
LMX 4	3.9 (0.8)	4.0 [3.0, 4.0]	3.8 (0.7)	4.0 [3.0, 4.0]	0.004
LMX 5	3.7 (0.8)	4.0 [3.0, 4.0]	3.4 (0.7)	3.0 [3.0, 4.0]	<0.001
LMX 6	3.6 (0.9)	4.0 [3.0, 4.0]	3.3 (0.8)	3.0 [3.0, 4.0]	<0.001
LMX 7	4.1 (0.7)	4.0 [4.0, 5.0]	4.1 (0.7)	4.0 [4.0, 5.0]	0.827
**SBQ-R Score**	3.7 (1.5)	3.0 [3.0, 4.0]	3.9 (1.8)	3.0 [3.0, 5.0]	0.045
SBQ-R 1	1.1 (0.4)	1.0 [1.0, 1.0]	1.1 (0.4)	1.0 [1.0, 1.0]	0.536
SBQ-R 2	1.1 (0.3)	1.0 [1.0, 1.0]	1.1 (0.5)	1.0 [1.0, 1.0]	0.712
SBQ-R 3	1.0 (0.2)	1.0 [1.0, 1.0]	1.1 (0.3)	1.0 [1.0, 1.0]	0.153
SBQ-R 4	0.5 (1.0)	0.0 [0.0, 0.0]	0.6 (1.1)	0.0 [0.0, 1.0]	0.030
History of Mental Illness (%)					0.329
No	467 (99.2)		825 (98.3)		
Yes	4 (0.8)		14 (1.7)		
Suicide Risk (%)					0.237
No	445 (94.5)		777 (92.6)		
Yes	26 (5.5)		62 (7.4)		

The Wilcoxon rank sum test was used to compare non-normal continuous variables; the chi-square test was used to compare categorical variables. SD, standard deviation; IQR, interquartile range. LMX, leader-member exchange; SFSS, subjective family socioeconomic status.

**Table 4:** Multiple regression models for SBQ-R scores in the male group and the female group.

Variable	Male	Female
Age	-0.023	0.017
	(-0.074, 0.028)	(-0.029, 0.064)
Program		
Master	Reference	Reference
Doctor	-0.391*	-0.143
	**(-0.717, -0.064)**	(-0.436, 0.150)
Marriage		
Yes	Reference	Reference
No	-0.191	0.034
	(-0.592, 0.210)	(-0.362, 0.431)
History of Mental Illness		
No	Reference	Reference
Yes	2.420***	2.584***
	**(1.028, 3.812)**	**(1.699, 3.470)**
LMX-7 Score	-0.081***	-0.109***
	**(-0.109, -0.053)**	**(-0.138, -0.080)**
SFSS	-0.028	-0.099*
	(-0.104, 0.047)	**(-0.176, -0.022)**
SFSS × LMX-7 Score	0.009	0.025**
	(-0.006, 0.024)	**(0.008, 0.041)**

β (95% CI). *p<0.05, **p<0.01, ***p<0.001. The bold indicates p<0.05. LMX, leader-member exchange; SFSS, subjective family socioeconomic status.

## 4 Discussion

In this study, we found significant associations between the risk of suicidal behaviors and student-supervisor relationship, as well as SFSS among medical graduate students. In addition, SFSS played a moderating effect in the association between the risk of suicidal behaviors and student-supervisor relationship. Among individuals with high SFSS scores, the association between suicide risk and student-supervisor relationships was weakened compared to those with low SFSS. Specifically, a significant association between suicide risk and poor student-supervisor relationships among medical graduate students was only observed when the SFSS was less than 7.82. The evidence gathered in this study has practical implications for medical educational institutions developing suicide prevention intervention programs based on these insights.

Suicidal ideation is prevalent in medical students, as they usually experience higher rates of depression, burnout, and mental disorders than the general population, especially in female medical students (2, 3). A recent study suggested that graduation pressure, depression, and academic pressure were the three leading suicidal causes in Chinese graduate students (23). In particular, medical graduate students face a number of academic, personal, and environmental challenges, and they have to deal with a roller-coaster of positive and negative emotions (11), which may put them at higher risk for suicidal behaviors. Therefore, identification of the associated factors with suicidal risk among medical graduate students can help the timely detection of the problem, and provision of appropriate interventions to prevent the suicidal behaviors.

Several previous studies have found that graduate students who have high-quality relationship with supervisors can receive effective academic and emotional support from their supervisors, thereby relieving mental stress and becoming more academically creative (10, 12, 24). However, graduate students without good student-supervisor relationships were likely to suffer from academic procrastination and lower subjective well-being (9, 11). In this study, a significant association between the risk of suicidal behaviors and student-supervisor relationships among medical graduate students was found by using LMX-7 questionnaire to evaluate the exchange of emotions and resources between superiors and subordinates. With a high LMX-7 relationship, graduate students can obtain academic and emotional support from their supervisor, which can help them significantly reduce challenge and hindrance stressors (8). On the contrary, a low LMX-7 relationship indicates reduced emotional support and less academic guidance from supervisors, which can lead to increased graduation pressure and higher suicide risk among medical graduate students.

Apart from student-supervisor relationship, socioeconomic status was considered as one determinant of suicidal behaviors in medical students (25, 26). SFSS assesses how people perceive their family’s position in the social hierarchy and has been shown to correlate with suicidality. For example, Chinese college students hailing from rural areas exhibited a significantly higher suicide risk compared to their urban counterparts (27). The current study used subjective measurements of family socioeconomic status to reflect the relative social status of students’ families, which was more effective than the objective measures and can capture its subtle aspects (28). In line with previous studies (14, 22, 29), our results demonstrate that a low SFSS was associated with an increased risk of suicide among medical graduate students, and it also moderated the association between the risk of suicidal behaviors and student-supervisor relationship. Medical graduate students from families with low SFSS may have taken on more financial pressure and family responsibilities, as well as have more aspirations for career advancement after graduation (30, 31). All these factors may collectively contribute to higher pressure, increased anxiety, and an elevated risk of suicidal behaviors.

As this special issue calls for, suicide prevention among medical graduate students is an urgent concern. Although medical students are often educated about mental health, studies have indicated that they tend to exhibit greater reluctance in seeking external assistance when facing psychological issues (32). Providing mental health training for medical graduate students that promotes seeking social support and psychological interventions for suicidal ideation and behavior could be advantageous. In addition, building a healthy student-supervisor relationship can be helpful for the fragile and high-pressure medical graduate students. The universities are suggested to establish professional courses for both students and supervisors to regulate the rights and obligations in their relationship (11). Moreover, it is necessary to pay attention to medical graduate students from families with low SFSS, as they face an elevated risk of suicidal behaviors in the context of subpar relationships with their supervisors. It is important to give more encouragement and guidance to these students to help them to communicate with supervisors more proactively and equally.

We acknowledged there are some limitations in this study. First, this cross-sectional study was not enough to determine the causal relationship between the risk of suicidal behaviors, student-supervisor relationship and SFSS. Future longitudinal studies are needed to confirm the causality of these associations and conclusions of this study. Second, this study only recruited graduate students from a single medical school, and generalization of the current findings needs to be verified by further multi-center studies.

## 5 Conclusion

The risk of suicidal behaviors was associated with student-supervisor relationships and SFSS among medical graduate students. Poor relationships with supervisor were associated with an elevated risk of suicidal, and SFSS moderated this association. Educators should pay increased attention to the suicidal risk among medical graduate students who have poor supervisor relationships, especially those from families with low SFSS. Providing regular psychological education and improving student-supervisor relationship may offer promising solutions for reducing the risk of suicidal behaviors among medical graduate students.

## Data availability statement

The raw data supporting the conclusions of this article will be made available by the authors, without undue reservation.

## Ethics statement

The studies involving humans were approved by West China School of Medicine/West China Hospital, Sichuan University. The studies were conducted in accordance with the local legislation and institutional requirements. The participants provided their written informed consent to participate in this study.

## Author contributions

YW: Conceptualization, Data curation, Investigation, Writing – original draft. ZQ: Conceptualization, Data curation, Writing – original draft, Writing – review & editing. WT: Conceptualization, Data curation, Writing – original draft. YZ: Formal Analysis, Writing – original draft. XX: Data curation, Writing – review & editing. ZY: Formal Analysis, Supervision, Writing – review & editing. ZL: Supervision, Writing – review & editing.
