# Supplementary material for: Reducing the risk of suicidal behaviors in medical graduate students: exploration of student-supervisor relationship and subjective family socioeconomic status
Source: Front Psychiatry. 2024 May 7;15:1381291. doi: 10.3389/fpsyt.2024.1381291 (PMC11106388; doi:10.3389/fpsyt.2024.1381291)
Supplement: Supplementary file 1 [file Table_1.docx]

***Supplementary Material***

**Suicidal Behaviors Questionnaire-Revised (SBQ-R) questionnaire**

**Instructions: Please check the number beside the statement or phrase that best applies to you.**

**1. Have you ever thought about or attempted to kill yourself in the past year?**

□ 1. Never

□ 2. lt was just a brief passing thought

□ 3a. I have had a plan at least once to kill myself but did not try to do it

□ 3b. I have had a plan at least once to kill myself and really wanted to die

□ 4a. I have attempted to kill myself, but did not want to die

□ 4b. I have attempted to kill myself, and really hoped to die

**2. How often have you thought about killing yourself in the past year?**

□ 1. Never

□ 2. Rarely (1 time)

□ 3. Sometimes (2 times)

□ 4. Often (3-4 times)

□ 5. Very Often (5 or more times)

**3. Have you ever told someone that you were going to commit suicide or that you might do it in the past year?**

□ 1. No

□ 2a. Yes, at one time, but did not really want to die

□ 2b. Yes, at one time, and really wanted to die

□ 3a. Yes, more than once, but did not want to do it

□ 3b. Yes, more than once, and really wanted to do it

**4. How likely is it that you will attempt suicide someday?**

□ 0. Never

□ 1. No chance at all

□ 2. Rather unlikely

□ 3. Unlikely

□ 4. Likely

□ 5. Rather likely

□ 6. Very likely

**Suicidal Behaviors Questionnaire-Revised (SBQ-R) questionnaire**

**Scoring guideline (total points range from 3 to 18)**

**Item 1**: response 1 (1 point), response 2 (2 points), response 3a or 3b (3 points), response 4a or 4b (4 points).

**Item 2**: response 1 (1 point), response 2 (2 points), response 3 (3 points), response 4 (4 points), response 5 (5 points).

**Item 3**: response 1 (1 point), response 2a or 2b (2 points), response 3a or 3b (3 points).

**Item 4**: response 0 (0 point), response 1 (1 point), response 2 (2 points), response 3 (3 points), response 4 (4 points), response 5 (5 points), response 6 (6 points).

**LMX-7 Questionnaire**

**Instructions: This questionnaire contains items that ask you to describe your relationship with your supervisor. Please evaluate the relationship between you and your supervisor based on your actual feelings and experiences. For each of the items, indicate the degree to which you think the item is true for you by ticking the most appropriate number to confirm.**

**1. How would you characterize your relationship with your supervisor?**

| Extremely  ineffective  1 | Worse than  average  2 | Average  3 | Better than  average  4 | Extremely effective 5 |
| --- | --- | --- | --- | --- |

**2. What are the chances that your supervisor would use his/her power and resource to help you solve your academic difficulties/find a job?**

| None  1 | Small 2 | Moderate  3 | High 4 | Very high  5 |
| --- | --- | --- | --- | --- |

**3. How well does your supervisor recognize your potential?**

| Not at all  1 | A little 2 | Moderately  3 | Mostly 4 | Fully  5 |
| --- | --- | --- | --- | --- |

**4. How well do you think your supervisor understands your academic problems and needs?**

| Not a bit  1 | A little 2 | A fair amount  3 | Quite a bit 4 | A great deal  5 |
| --- | --- | --- | --- | --- |

**5. How satisfied do you think your supervisor is with your academic performance?**

| Very dissatisfied 1 | Dissatisfied 2 | Neutral  3 | Satisfied 4 | Very satisfied  5 |
| --- | --- | --- | --- | --- |

**6. What are the chances that your supervisor would help you out of academic difficulties at his/ her own expense?**

| None  1 | Small 2 | Moderate  3 | High 4 | Very high  5 |
| --- | --- | --- | --- | --- |

**7. I have enough confidence in my supervisor that I would defend and justify his/her decision if he/she were not present to do so.**

| Strongly disagree  1 | Disagree 2 | Neutral  3 | Agree 4 | Strongly agree  5 |
| --- | --- | --- | --- | --- |

**Subjective family socioeconomic status**


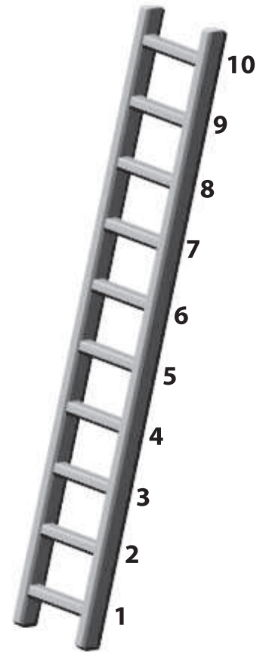


Think of this ladder as representing different social classes of different families in China. At the top of the ladder (step 10) are the people who have the best living conditions. They are highly educated, have the most decent jobs, and have the highest incomes. At the bottom of the ladder (step 1) are the people who have the worst living conditions, with the lowest education level, the least decent jobs, and the lowest incomes.

**Where would you place your family on this ladder?** Please select the number for the step that shows where you think your family stands relative to other families in China.
